# Muscle Proteins, Technological Properties, and Free Amino Acids of Epaxial Muscle Collected from Asian Seabass (*Lates calcarifer*) at Different Postmortem Durations

**DOI:** 10.3390/ani14192837

**Published:** 2024-10-01

**Authors:** Yuwares Malila, Danai Charoensuk, Yanee Srimarut, Sunitta Saensa-ard, Nalinrat Petpiroon, Chanikarn Kunyanee, Wachiraya Rattanawongsa, Rattaporn Saenmuangchin, Annop Klamchuen, Wiyong Kangwansupamonkon, Sasitorn Aueviriyavit

**Affiliations:** 1 Food Biotechnology Research Team, National Center for Genetic Engineering and Biotechnology (BIOTEC), 113 Thailand Science Park, Khlong Nueng, Khlong Luang, Pathum Thani 12120, Thailand; danai.cha@biotec.or.th (D.C.); yanee.sri@biotec.or.th (Y.S.); 2 National Nanotechnology Center, National Science and Technology Development Agency, 111 Thailand Science Park, Khlong Nueng, Khlong Luang, Pathum Thani 12120, Thailand; sunitta.sae@ncr.nstda.or.th (S.S.-a.); nalinrat.pet@nanotec.or.th (N.P.); chanikarn.kun@nanotec.or.th (C.K.); wachiraya.rat@ncr.nstda.or.th (W.R.); rattaporn.sae@nanotec.or.th (R.S.); annop@nanotec.or.th (A.K.); wiyong@nanotec.or.th (W.K.); 3 AFRS(T), The Royal Society of Thailand, Sanam Sueapa, Dusit, Bangkok 10300, Thailand

**Keywords:** Asian seabass, muscle protein, rigor mortis, animal welfare

## Abstract

**Simple Summary:**

The eating quality of fish is considered best when the durations between the slaughter, cooking, and consumption of the fish are minimized. Such a perception has raised animal welfare issues, as fish are captured, potentially under stress, before slaughter. In this study, it was revealed that with proper storage handling (in an ice box; ratio of fish to ice = 1:1; 2 h ice refills), the epaxial muscle samples of Asian seabass, one of the most economically important fish species, collected 24 h postmortem exhibited a greater degree of water-holding capacity and a more tender texture than 1 h postmortem samples. Free amino acids related to umami and sweetness were increased in the cooked samples collected 24 h postmortem. At 24 h postmortem, although the structural proteins of the epaxial muscles of Asian seabass remained intact, the myofibrils had undergone fragmentation to a greater extent, corresponding to meat tenderization without negative impacts on the springiness and cohesiveness of the cooked meat.

**Abstract:**

The aim of this study was to compare the changes in the epaxial muscle proteins of Asian seabass at two different postmortem durations. The epaxial muscles of Asian seabass were collected 1 h or 24 h postmortem (PM). Whole, ungutted fish were stored in an ice box, with the ice refilled every two hours. The results show significant increases in the MFI values and the contents of solubilized sarcoplasmic proteins, with a molecular weight of proteins of 47 kDa in the 24 h PM samples (*p* < 0.05). Myofibrillar and alkaline-soluble proteins in the epaxial muscle remained intact 24 h postmortem. Compared with the 1 h PM samples, the 24 h PM meat exhibited lower degrees of expressible water and hardness (*p* < 0.05), indicating superior water-holding capacity and meat tenderness. However, no differences in springiness or cohesiveness of the cooked meat were observed. Free L-glutamic acid, known as an umami-tasting amino acid, was significantly increased upon the extension of postmortem duration, and its level was above the taste threshold concentration. Overall, the findings indicated that the 24 h PM epaxial muscle of Asian seabass exhibited superior technological properties, along with higher contents of taste-related amino acids.

## 1. Introduction

Asian seabass, or barramundi (*Lates calcarifer*), is an important food fish species belonging to the order of Perciforms [1]. Widely found in the Indo-Pacific region, Asian seabass has been successfully cultured since the 1970s in Thailand and was later introduced to Southeast Asian and other countries, including Taiwan, Hong Kong, China, Saudi Arabia, and Australia [1]. Today, the fish are generally either obtained from aquaculture or are wild-caught and are distributed as live or fresh fish [2]. Live fish are held in fish tanks or small ponds at fresh markets or restaurants and immediately slaughtered before being sold and cooked. Unlike live fish, fresh fish are caught alive, either with complete organs or gutted and dressed, immediately followed by chilling to preserve freshness [2]. In Asian countries, many consumers prefer live fish over fresh fish, including Asian seabass, that is, fish that are immediately slaughtered before consumption [3]. This is due to post-rigor muscle softening, which consumers often associate with the perception that the fish is no longer fresh and tastes bland [4,5]. As a result, such perceptions have led to the consideration of fish as sentient beings. Live fish are usually kept alive at high stocking densities, initiating potential pre-slaughter stress [6]. Such practices, along with a low-tech slaughtering process, are often concerns raised with respect to potential animal welfare issues [6,7].

Recently, there has been an interesting shift in the culinary landscape towards aged fish [3]. This is due to the release of flavor-giving short peptides and potential bioactive compounds during postmortem protein degradation [3,8]. A study conducted by Minami et al. [9] showed increased tenderness and increased contents of amino acids related to umami and the sweet taste in fish and shellfish subjected to long-term aging at 1 °C. In addition, Panebianco et al. [3] reported that microbial loads in gutted rainbow trout subjected to a dry aging at 2 °C and a relative humidity of 78% remained at acceptable levels for 10 days. Furthermore, when pre-slaughter stress is minimized, the technological properties and eating quality of fish flesh are less likely to be negatively impacted by muscular energy exhaustion and muscle acidification [10].

Similar to mammals, the three postmortem phases, i.e., the pre-rigor, rigor, and post-rigor stages, can influence the flesh quality of fish. Shortly after slaughter, the metabolic process of muscle cells is switched from oxidative respiration to anaerobic glycolysis for ATP production. Eventually, ATP becomes inadequate for detaching the actomyosin complex, initiating rigor mortis, i.e., the stiffening of the muscles. Without blood circulation, lactate accumulation lowers the pH of the muscle. The ultimate pH depends on the species, as well as the pre- and post-slaughter conditions. The magnitude of the final pH and the rate of pH decline are associated with the properties of the muscle protein; therefore, they mainly influence visual characteristics, water-holding capacity and texture of the meat [11]. The onset and duration of rigor differ depending on the species and the condition of animals. In general, in small fish, pre-slaughter stress causes muscle glycogen depletion, and improper postmortem handling (e.g., a high storage temperature) can accelerate the rigor mortis [6]. As a consequence of the decline in pH, the cells cannot maintain their physiological conditions, leading to the release of calcium ions (Ca^2+^), which later trigger the proteolytic autolysis of the muscle [12]. The primary endogenous proteases playing roles in postmortem muscle fiber degradation include calpains, with the further involvement of calpastatin, cathepsins, caspases, and proteasomes [13]. The proteases break down myofibrils but do not detach the actomyosin complex, leading to the fragmentation of myofibrils, resulting in meat tenderization [12,13]. In addition, the activities of the endogenous enzymes further release free amino acids and free fatty acids, providing taste and aroma [14].

Postmortem biochemical phenomena vary due to several antemortem factors, including species, age, diet, and pre-slaughter conditions. Pre-slaughter stress in animals can be induced when they experience physiological and emotional difficulties and are not able to adapt to such constraints [15]. Such stress can lead to increases in heart rate and respiration, initiating energy production and other biochemical processes [16] and thereby affecting meat quality. In aquatic animals, stress may be related to feeding, water quality, capture, and the pre-slaughter period [17]. Lippe et al. [18] reported that the myosin light chain in the fillets of pre-slaughter starved seabream had undergone proteolysis to a greater extent than that of fully fed samples. Recently, the influences of pre-slaughter stunning methods were examined [6]. The results showed that an application of electrical pre-slaughter stunning induced pre-slaughter stress in *Arapaima gigas* in comparison to the conventional ice asphyxiation. The fillet of the former group also exhibited a greater integrity during 15 h storage under refrigerated conditions. In contrast, the study of Castro et al. [17] addressed the resistance against pre-slaughter hypoxia (0, 3, and 6 min) among Nile tilapia, along with no significant impacts shown on the meat quality during a 10-day storage period.

Previous studies have also addressed the activation of the endogenous proteases in European seabass (*Dicentrarchus labrax*) [19,20] due to the postmortem pH decline and increased intramuscular ionic strength [8]. The cytosolic and lysosomal proteases degraded the structural muscle proteins, leading to disorganization of the Z-lines and detachment of the sarcolemma [8,19,20]. However, while myofibril-associated proteins are the primary targets of postmortem proteolytic processes in mammals, the actomyosins of the European seabass remained intact up to 14 days at 4 °C storage [19]. In addition, the activity of the cathepsins in the white muscle of the European seabass remained steady during storage at 4 °C for up to 7 days postmortem [20]. The large proteins (i.e., titin, nebulin, and dystrophin) of the seabass were markedly degraded, along with its sarcolemma, following 2 days of cold storage [19].

Despite its economic value, the information regarding postmortem protein changes in Asian seabass is still scarce. The objective of this study was to characterize the postmortem protein changes, associated with meat quality indices, at different postmortem durations. The technological properties of Asian seabass were also determined. Two postmortem durations (i.e., 1 h and 24 h postmortem) were chosen to represent the changes during pre- and post-rigor, respectively. The obtained results supported no significant advantages of the pre-rigor meat quality which encouraged the humane operation of Asian seabass.

## 2. Materials and Methods

### 2.1. Samples and Sample Collection

**Figure 1:**
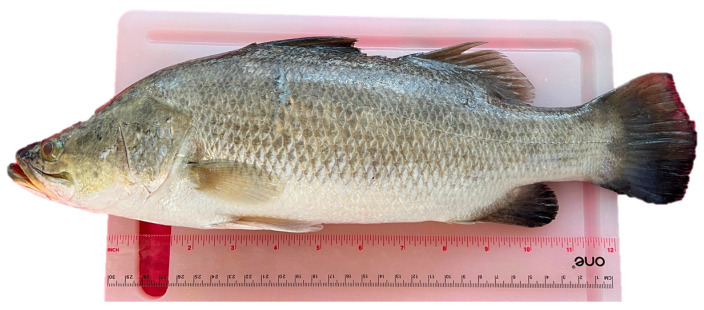
Asian seabass (*Lates calcarifer*) with approximate live weight of 800 g.

A total of 10 live 8-month-old Asian seabass (*Lates calcarifer*) with an approximate live weight of 800 g (Figure 1) were obtained from a local market (Pathum Thani, Thailand). Whole, ungutted fish were immersed in ice at a ratio of fish to ice equal to 1:1 by weight. Each fish was packed in a Styrofoam box and transported to the laboratory (Nano Safety and Bioactivity Research Team, Pathum Thani, Thailand) which took about 10 min driving from the market. Upon arrival, the fish were euthanized by an immersion in water containing adequate dosage of clove oil for 10 min to ensure its euthanasia stage. The fish body surface was then washed and disinfected by submersion in 70% ethanol for 30 s prior to scaling, tail-cutting, bleeding, and gutting. The fish were then divided into two groups. The first group, labeled as “1 h postmortem (1 h PM)”, were immediately washed and filleted. Epaxial muscles were dissected, deskinned, weighed, and immediately proceeded for determining myofibril fragmentation index (MFI) and muscle protein composition. The remaining epaxial muscle was vacuum-packed in a polyethylene bag and frozen at −20 °C for meat quality determination. The other five fish were stored in the ice box until reaching 24 h postmortem. The ice was refilled every two hours to maintain the temperature of 0 °C to 3 °C during the storage. The ratio of fish to ice was 1:1 by weight. The samples were then prepared in a similar manner as described for 1 h PM samples but were labeled “24 h PM”. All steps were performed at temperature of 0 °C to 4 °C.

All experimental protocols of this study were reviewed and approved by the Institutional Animal Care and Use Committee of National Science and Technology Development Agency (project ID 002-2567, 1 April 2024).

### 2.2. Myofibril Fragmentation Index

Myofibril fragmentation index (MFI) of the samples was analyzed according to Trithavisup et al. [21] with a slight modification. In brief, 0.5 g of raw fish samples was homogenized at the speed of 9600 rpm in 30 mL of ice-cold MFI buffer (25 mM potassium phosphate buffer, a pH of 7.0, containing 0.1 M KCl, 1 mM EDTA, and 1 mM sodium azide) using an ULTRA-TURRAX T25 Basic homogenizer (IKA Werke, Staufen, Germany). Homogenization was performed for 2 min with two cycles of 30 s on and 30 s rest. The homogenate was then centrifuged at 1000× *g*, 4 °C, for 10 min. The supernatant was discarded, and the pellet was homogenized with 20 mL of cold MFI buffer under similar aforementioned condition. The final pellet was resuspended in 10 mL of cold MFI buffer. Protein concentration of the myofibril suspensions was examined following bicinchoninic acid assay. Absorbance at 540 nm of the suspension, diluted to a 2 mL aliquot with protein concentration of 0.5 mg/mL, was measured. MFI buffer was used as a blank. MFI value was calculated by multiplying the average absorbance with 150. 

### 2.3. Muscle Protein Composition

Muscle proteins, namely sarcoplasmic (SP), myofibrillar (MP), and alkaline-soluble (AS) proteins, were extracted according to their different solubilities. Half a gram of the sample was homogenized, on ice, with 5 mL of 20 mM Tris-HCl buffer (pH of 7.5) supplemented with SIGMAFAST™ protease inhibitors (Sigma-Aldrich Co. LLC, St. Louis, MO, USA). Using an ULTRA-TURRAX T25 Basic homogenizer (IKA Werke, Staufen, Germany) at the speed of 13,600 rpm, the homogenization was carried out for 2 min with six cycles of 10 s on, followed by 10 s rest. The homogenate was then centrifuged at 5000× *g*, 4 °C, for 15 min. The supernatant was collected, weighed, and labeled as “SP”. The resulting pellet was subsequently homogenized with 5 mL of 20 mM Tris-HCl buffer (pH 7.5), containing 0.6 M KCl and the protease inhibitors. The homogenization and centrifugation were conducted in a similar manner to the SP extraction. The resulting supernatant was weighed and labeled as “MP”. The remaining pellet was solubilized in 5 mL of 5% sodium dodecyl sulfate (SDS) at 80 °C for 60 min, followed by a centrifugation at 5000× *g* for 15 min at ambient temperature. The supernatant was collected, weighed, and labeled as “AS”. Protein concentration of SP, MP, and AS was determined using a bicinchoninic acid (BCA) assay. Bovine serum albumin was used for standard curve preparation. The amount of muscle proteins was expressed in mg protein per g sample, and later converted into the ratio of SP to MP to AS.

### 2.4. Sodium Dodecyl Sulfate–Polyacrylamide Gel Electrophoresis (SDS-PAGE)

Electrophoretic patterns of the extracted SP, MP, AS, and SDS samples were analyzed using an SDS-PAGE. As for the SDS-extracted samples, 0.5 g of meat sample was homogenized with 5 mL of 5% SDS solution using an ULTRA-TURRAX T25 Basic homogenizer (IKA Werke, Staufen, Germany) set at the speed of 13,600 rpm and incubated at 80 °C for 60 min. After a centrifugation at a speed of 5000× *g* for 15 min at ambient temperature, the supernatant was collected and labeled as “SDS-extract”. The concentration of the extracts was then determined using a BCA assay.

To perform SDS-PAGE, 20 µg of protein samples was mixed with 2 µL of 5X sample buffer, containing 50% glycerol, 10% SDS, 0.5% bromophenol blue, 250 mM Tris-HCl, a pH of 6.8, and 20% beta-mercaptoethanol. The final volume of the mixture was adjusted to 10 µL using deionized water, followed by an incubation at 95 °C for 5 min. The protein mixture was then loaded into a 4–20% Mini-PROTEAN TGX stain-free gel (Bio-Rad Laboratories, Richmond, CA, USA). Tris/glycine/SDS buffer (25 mM Tris, 192 mM glycine, 0.1% SDS, pH of 8.3) and Precision Plus Protein^TM^ Unstained Protein Standards with molecular weight of 10–250 kDa (Bio-Rad Laboratories, Richmond, CA, USA) were used as a running buffer and a molecular weight marker, respectively. Electrophoresis was conducted at a constant 120 V for 75 min. The gel was then scanned under a UV transillumination mode using a Gel Doc™ XR+ imaging system (Bio-Rad Laboratories, Richmond, CA, USA). Images and band volume (i.e., the sum of intensities within band boundaries) were acquired using an Image Lab™ software version 5.1 (Bio-Rad Laboratories, Richmond, CA, USA). Band volume was automatically converted into % lane (i.e., percentage of the band’s volume compared to the entire volume of the lane) by the software and used for further statistical analysis.

### 2.5. Thawing and Cooking Loss

Frozen fish fillets were thawed at 4 °C overnight. Thawing loss was expressed in percentage as different weights before and after thawing. Subsequently, the samples were cooked using a water immersion method [22] with a slight modification. In brief, each sample was vacuum-packed in a polyethylene bag and incubated at 95 °C for 10 min. The meat was subsequently cooled down in an iced water bath for 5–7 min. After the sample was left to rest on ice for 30 min, the sample was reweighed. Cooking loss was calculated and reported as the difference, as a percentage, between the weights before and after the meat was cooked.

### 2.6. Surface Color

Surface color in CIE L*, a*, and b* system of the sample was determined in both raw and cooked samples using a Minolta colorimeter (model CR300, Minolta Co., Ltd., Osaka, Japan). The meat was left to bloom on ice for 30 min before the determination.

### 2.7. pH

The pH of the samples was examined in raw and cooked samples using a spear-shaped pH probe (Mettler-Toledo Seven Easy, Mettler-Toledo, Inc., Greifensee, Switzerland).

### 2.8. Expressible Water

Expressible water of the cooked samples was determined using a TA-XTi texture analyzer (Stable Micro Systems, Godalming, UK) equipped with a 50 mm cylindrical aluminum probe. The samples, cut into cubes (10 mm × 10 mm × 10 mm), were placed between two pieces of Whatman No. 1 filter paper (Whatman International Ltd., Maidstone, UK). The samples were then subjected to a compression at 70% strain for 1 min. The direction of compression was perpendicular to the myoseptum. Differences in sample weight before and after the compression were used in the calculation of expressible water using the following equations:

$$Expressible\;water\;\left(\%\right)=\frac{\left({wt}_{i}-{wt}_{f}\right)}{{wt}_{i}\times MC}$$

where ${wt}_{i}$ = weight before compression; ${wt}_{f}$ = weight after compression; and *MC* = moisture content.

### 2.9. Texture Profile Analysis (TPA)

The cooked sample was cut into six cubes (10 mm × 10 mm × 10 mm). Each cube was then subjected to a texture profile analysis using a TA-Xti texture analyzer (Stable Micro Systems, Godalming, UK). The cooked meat was double-compressed perpendicular to the myoseptum using a 50 mm cylindrical aluminum probe. The test condition was set at 1 mm/s probe velocity, holding for 1 s, 0.1 N trigger force, and 40% strain. TPA parameters were calculated by the Exponent software version 6.1.11.0 (Stable Micro Systems).

### 2.10. Free Amino Acids

Free amino acids in cooked samples were analyzed using a gas chromatography–mass spectrometry (GC-MS) following the method previously described [23]. In brief, 400 mg of the cooked samples were sonicated with 4 mL of 25% acetonitrile in 0.1N HCl for 20 min. The sample was centrifuged at 9000 rpm for 20 min. Supernatant (150 µL) was then mixed with 50 µL norleucine and dried using a concentrator (Concentrator plus, Eppendorf, Hamburg, Germany) for 2 h. Fifty microliters of N-tert-butyldimethylsilyl-N-methyltrifluoroacetamide containing 1% tert-butyldimethyl chlorosilane (Sigma-Aldrich, St. Louis, MO, USA) and 50 µL acetonitrile were then added. The mixture was incubated at 100 °C for 4 h. One microliter of the derivatized sample was injected, following a spitless mode, onto a GC 7890A/MS 5975C System (Agilent Technologies, Palo Alto, CA, USA) equipped with a DB-5 column (50 m × 0.25 mm i.d., 0.1 µm, Agilent Technologies, Palo Alto, CA, USA). Carrier gas was helium adjusted at a flow rate of 1.2 mL/min. The GC condition was set for a stepwise temperature increment from an initial temperature of 170 °C (hold for 5 min) to 200 °C (ramp rate of 4 °C/min, then hold for 3 min), and a final temperature of 285 °C (ramp rate of 4 °C/min). The MS condition was set as follows; ion source of 230 °C, ionization energy of 70 eV, and mass range from 35 to 800 *m*/*z*. Free amino acid content, expressed in mg/100 g sample, was calculated using standard curves created from a mixture of the standard amino acid solution.

### 2.11. Statistical Analysis

Statistical differences between 1 h PM and 24 h PM were determined using Student’s *t*-test where the significant difference was set at 95% confidence.

## 3. Results

### 3.1. Myofibril Fragmentation Index (MFI)

**Figure 2:**
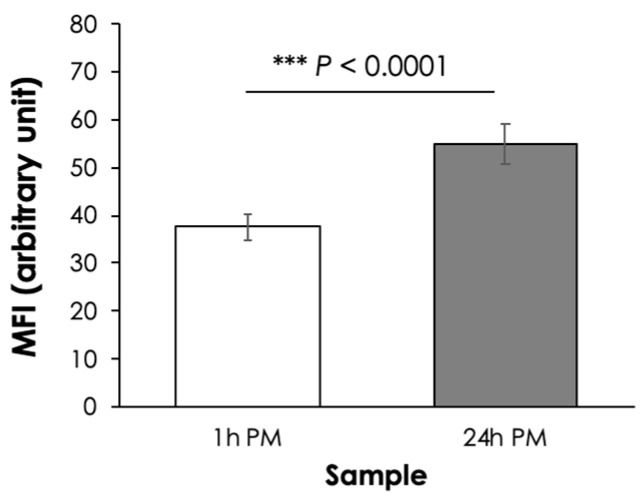
Myofibril fragmentation index (MFI) of epaxial muscle collected from Asian seabass with at 1 h and 24 h postmortem (PM). Bars and error bars represent mean and standard deviation, respectively. *** *p* < 0.001 indicates significant difference calculated using Student’s *t*-test.

In this study, the 24 h PM samples showed a greater MFI value than that of 1 h PM sample (Figure 2). The greater MFI of the 24 h PM samples suggested that its myofibrils had undergone fragmentation to a greater extent, hence the increased tenderness, than those of the 1 h PM samples. The current results agreed well with the previous study of Caballero et al. [24], in which increased muscle ultrastructural changes in sea bream (*Sparus aurata*) were addressed with longer postmortem refrigerated storage [25].

### 3.2. Muscle Protein Composition

**Figure 3:**
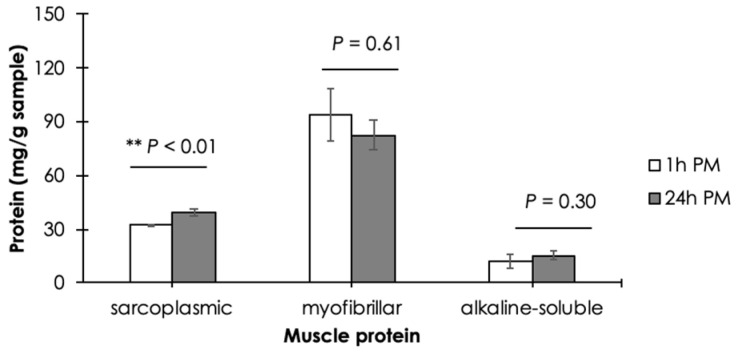
Ratio of muscle proteins, including sarcoplasmic (SP), myofibrillar (MP), and alkaline-soluble (AS) proteins, extracted based on solubilities from epaxial muscle of Asian seabass at 1 h and 24 h postmortem (PM). Bars and error bars represent mean and standard deviation, respectively. ** *p*-values above bars were calculated using Student’s *t*-test to identify statistical difference between 1 h PM vs. 24 h PM samples.

The effects of postmortem duration on the content of soluble SP, MP, and AS extracted from Asian seabass epaxial muscle are shown in Figure 3. No significant differences in MP and AS were observed between the 1 h PM and 24 h PM samples (*p* ≥ 0.05). The results of MP suggested that the conformational structures of Asian seabass epaxial MP were still preserved at 24 h PM. Therefore, the results led to the assumption of a comparable water-holding capacity of the MP between the two samples. However, as the postmortem duration was extended, the content of soluble SP was significantly increased (*p* < 0.05).

### 3.3. Protein Electrophoresis

**Figure 4:**
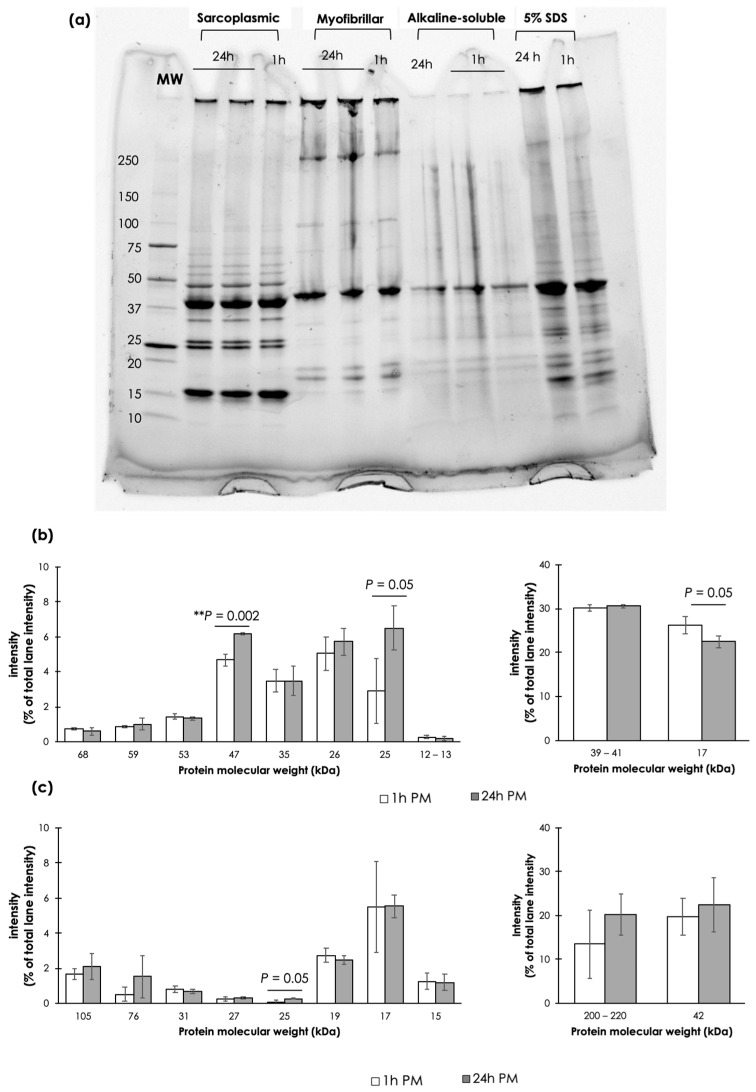
Effects of postmortem duration on electrophoretic patterns of proteins using an SDS-PAGE. (**a**) An example of SDS-PAGE of sarcoplasmic, myofibrillar, and alkaline-soluble proteins, extracted from epaxial muscle of Asian seabass with at 1 h and 24 h postmortem (PM). Twenty micrograms of proteins were loaded on the 4–20% gel. (**b**) Band intensity of sarcoplasmic proteins with molecular weight ranging from 12 to 60 kDa. (**c**) Band intensity of myofibrillar proteins with molecular weight ranging from 15 to 220 kDa. Bars and error bars represent mean and standard deviation, respectively, of five replicates. *p*-values above bars were calculated using Student’s *t*-test to identify statistical difference between 1 h PM vs. 24 h PM samples. ** *p* < 0.01.

Electrophoretic patterns of the extracted SP, MP, AS, and SDS proteins on 4–20% SDS-PAGE are shown in Figure 4a. On the SDS-PAGE, SP and MP of both samples comprised about 10 apparent bands with an average molecular weight of the proteins ranging from 68 to 12, and 220 to 15 kDa, respectively. The observed SP bands corresponded with the heme proteins (i.e., hemoglobin; 68 kDa, and myoglobin; 17 kDa), glycolytic enzymes (e.g., pyruvate kinase; 68 kDa, beta-enolase; 57 kDa, aldolase; 40 kDa, fructose-bisphosphate aldolase A; 39–40 kDa, glyceraldehyde phosphate dehydrogenase; 35 kDa, and triosephosphate isomerase; 26 kDa), other cytosolic proteins (phospholipase; 13 kDa, and parvalbumin; 12 kDa), and supplementary protein band (25 kDa) [26]. As for MP bands, the corresponding proteins included myosin heavy chain (200–220 kDa), alpha-actinin (105 kDa), troponin (76 kDa), actin (42 kDa), sub-troponin (31 kDa), and myosin light chains (27, 19, and 15 kDa) [27]. Two more MP bands, with unknown corresponding proteins, were observed at 17 and 25 kDa. The AS fraction showed apparent bands at molecular weights of 40–45, 30, and double bands at 18–20 kDa. Focusing on SDS-extracted samples, 5% SDS could solubilize most of the proteins in the muscle samples; therefore, the band patterns of the SDS-extracted samples showed a combination of the bands found among SP, MP, and AS samples. The resulting patterns are similar to those of fish muscles previously reported [5,28,29,30].

Considering the SP patterns (Figure 4b), the 24 h PM samples showed significant increases in the band intensity of the SP with a molecular weight of 47 kDa with respect to that of 1 h PM. Despite no significant differences (*p* ≥ 0.05), the intensity of the band detected at 25 kDa in the 24 h PM samples tended to increase, whereas the intensity of the band at 17 kDa tended to decrease. The current densitometric quantification of the SP band patterns agreed well with the increased MFI and SP content, indicating smaller molecules of water-soluble oligopeptides potentially broken down from myofibril fragmentation and protein autolysis. Such changes would promote a tender texture of the 24 h PM epaxial samples. On the other hand, most of the MPs did not show any significant changes in band intensity between the 1 h PM and 24 h samples.

### 3.4. Technological Properties

As shown in Table 1, raw samples of 24 h PM showed a significantly increased pH, L*-value, and b*-value (*p* < 0.05). Considering the cooked samples, the a*-value was increased while the expressible water and hardness were decreased in the 24 h PM than in those of the 1 h PM samples (*p* < 0.05). The results indicated that in raw epaxial muscle, an extended PM duration from 1 h to 24 h PM contributed to an increased meat alkalinity and deviated visual characteristics (i.e., increased lightness and decreased blueness of surface color). On the other hand, as the postmortem duration was extended, the surface color of the cooked meat became redder. Meanwhile, the expressible water and hardness of the cooked meat were reduced. The findings in the cooked samples indicated that the 24 h PM meat exhibited juiciness and tenderness to a greater extent than those of the 1 h PM samples, corresponding well with the greater MFI and comparable MP solubility.

### 3.5. Free Amino Acids in Cooked Sample

The free amino acids of the cooked epaxial muscles of Asian seabass collected at different postmortem timepoints are shown in Table 2. In this study, the focus was only on amino acids related to the taste profiles of foods. The results showed that the majority of the free amino acids exhibited greater contents in the 24 h PM samples compared to their counterparts (*p* < 0.05), except for proline, serine, and histidine. Such increased content of the free amino acids supported the assumption of increased SP due to further proteolytic degradation. Meanwhile, the contents of the sweet-tasting amino acids were reduced in the 24 h PM samples by 13%, and the contents of the umami-tasting and bitter-tasting amino acids were increased in the 24 h PM samples by 28% and 53%, respectively. Considering mainly the taste threshold [31], only L-glutamic acid content exceeded the taste threshold. Based on the current findings, it could be speculated that the umami flavor of the 24 h PM samples would be stronger than that of the 1 h PM ones.

## 4. Discussion

The meat quality and sensory characteristics of muscle-based foods are greatly dependent on the biochemical processes that occur in the non-living stage of the animals. The main objective of this study was to explore the postmortem protein changes, which were related with the meat quality of the epaxial muscle of Asian seabass at pre- and post-rigor stages. In general, the live Asian seabass immediately slaughtered at the restaurant or fresh markets are more favorable among Asian consumers. This is due to the perception that the immediately slaughtered fish exhibited better texture, taste, and flavor. However, the captivation of the live fish in small containers as well as an inhumane operation would create pre-slaughter stress, negatively impacting meat quality [6]. It is worth noting that as the aim of this study was to compare pre- and post-rigor samples, the pre-rigor samples should be collected immediately after slaughter. However, based on the sample collection process (i.e., slaughtering, bleeding, and sample dissection) which took almost 1 h to complete, the representatives of pre-rigor samples were labeled as 1 h PM to truly represent its actual PM duration.

Following death, anaerobic glycolysis becomes the main biological energy-production process through the conversion of glycogens into glucoses, ATPs, and lactates. As blood circulation and waste removal are terminated, lactate is accumulated within the muscles, resulting in pH decline. The rate of pH decline and the ultimate pH generally influence technological properties of meat proteins. In this regard, it was initially assumed that pH of the 24 h PM samples would be either comparable or lower than that of 1 h PM muscles. However, the obtained results of a slightly, but significantly, higher pH of the 24 h PM samples might be an implication of the higher initial glycogen content in the 24 h PM muscle [32]. The results appeared to support a greater degree of pre-slaughter stress within the 1 h PM samples although, under this study, the fish were subjected to a pre-slaughter stunning using clove oil. A similar issue was addressed by Poli et al. [10], that fish captivation may induce stress, contributing to energy exhaustion and muscle acidification. Generally, the increased pH of the fish reflected the decreased freshness of the fish. In this regard, an additional speculation could be that the high pH currently observed in the 24 h PM samples was due to bacterial activity that decomposed the nitrogenous compounds at the post-rigor state. As a consequence, the muscle pH was increased from an acidic to alkaline range [32].

In fish, the onset of rigor mortis was accelerated when the difference in temperature between the fish habitat and the storage ones was large [33]. At the post-rigor stage, an autolysis of proteins occurred. During this process, the structural components, including myofilaments and connective tissues, of the fish muscle were degraded, leading to the muscle tenderization. In this study, as expected, the greater MFI value was observed for the epaxial muscle collected at the longer PM period. The results indicated an increased tenderness as the postmortem duration was extended. In general, the MFI value is an indicator of the postmortem proteolytic breakdown of the I-band and intermyofibrillar linkages [34,35]. An increased MFI value agreed well with the decreased hardness in the 24 h PM cooked samples. Nonetheless, as no significant differences in springiness and cohesiveness of the cooked meat (*p* ≥ 0.05) were observed, the findings indicated that an increased meat tenderness due to the myofibril fragmentation at 24 h PM did not negatively affect the molecular cohesion within the cooked meat. The meat remained intact. Furthermore, an increase in myofibril fragmentation in the 24 h PM samples might contribute to an increased light scattering on the meat surface [30], leading to the lighter surface color of the 24 h PM samples. In general, as the postmortem storage duration was extended, oxymyoglobin in the fish muscle underwent oxidation. Hence, ferrimyoglobin, a brownish yellow pigment, was formed. Such biochemical changes would cause a reduction in the a*-value and an increase in the b*-value in the raw fish meat [36]. In this study, raw samples showed no significant changes in the a*-value and a slight decrease in the b*-value. Although further investigation is required, it might be speculated that the current postmortem durations might exert only a subtle effect on the color of the fish muscles.

Protein solubility is often investigated to determine the technological properties of the meat. In this study, the ratio of the extracted SP:MP:AS from both samples was approximately 30:50–60:10 which agreed well with the muscle protein compositions [11]. In agreement with the current results, the structural proteins, including actin and desmin, were persistent up to 14- and 10-day postmortem in the muscle of European seabass [19] and sea bream [24,25], respectively. The skeletal muscle of farmed Atlantic salmon also showed a low susceptibility against autolysis up to 48 h postmortem when the fish was stored at 4 °C [37]. As for AS, the total collagen, the main composition of AS, was not changed in sea bream during 24 h postmortem storage either at 1 °C or 4 °C [28]. Overall, the results indicated that, apart from SP, the postmortem extension in this study did not significantly affect the MP solubility. The technological properties of the Asian seabass epaxial MP at 24 h PM appeared to be preserved in cold storage. The protein patterns found on the electrophoresis corresponded well with the results of protein solubility. The most intensive SP band was at 39–41 kDa which potentially corresponded with fructose–-bisphosphate aldolase A and aldolase [5]. A significant increase in the SP band intensity at 47 kDa, along with a trending increase in the intensity of protein at 25 kDa, which was considered a supplementary band, indicated the breakdown of high molecular weight proteins. Nonetheless, the Asian seabass epaxial SP, MP, and AS did not showed drastic changes in electrophoretic patterns between the 1 h PM and 24 h PM groups. The findings suggested that although the texture of the 24 h PM cooked meat tended to be more tender, the overall structure of the meat remained intact with superior technological properties. Similar results were observed in European seabass. Ladrat et al. [5] reported the stability of such proteins, which were isolated from European seabass, against cathepsins isoform B, D, and L within the first 2 h of incubation at 25 °C. Also studied in European seabass, Verrez-Bagnis et al. [38] found a gradual disappearance of SP at 17 kDa, corresponding to nucleoside diphosphate kinase, within 24 h cold storage. In contrast, Suarez et al. [28] addressed the rapid degradation of dystrophin, a 427 kDa sarcolemmal actin-binding protein, during the first 24 h postmortem and completely disappeared within 2–3 days. It could be speculated that the trend of reduced MP and increased SP in the 24 h PM samples might be attributed to the degradation of dystrophin into peptides, which were able to be solubilized in a low-ionic strength buffer. However, dystrophin molecular size was too large to be detected on the current SDS-PAGE.

To determine whether an extension of postmortem duration would provide any potential effects on the taste of the Asian seabass epaxial muscle, free amino acids were profiled using a GC-MS. Compared with the 1 h PM samples, the contents of several amino acids were greater in the 24 h PM epaxial samples. The current results were expected and corresponded well with the increased SP solubility as well as the observed electrophoretic patterns of the 24 h PM samples. The taste-active amino acids could be released from muscle protein breakdown by endogenous proteases (e.g., cathepsin B) [39]. Based on the results, the total content of the sweet-tasting amino acids was decreased as the postmortem duration extended. In contrast, the total contents of the umami-tasting and bitter-tasting amino acids increased. Based on the current results, the cooked meat of the 24 h PM samples could taste less sweet with more umami flavor and more bitterness. However, only the content of L-glutamic acid, the umami-tasting amino acid, exceeded the taste threshold concentration [31]. Although the sensory evaluation remained to be further investigated, the current results imply that the cooked 24 h PM meat provided umami taste to a greater extent compared with that of the 1 h PM samples. The overall findings of this study indicated that the meat of live Asian seabass immediately slaughtered before consumption might not always provide a superior eating quality than that of fresh fish stored on ice. The practice of fish capture could be omitted for the better welfare of Asian seabass [6]

## 5. Conclusions

In this study, the postmortem changes in muscle proteins, technological properties, and free amino acids of epaxial muscle collected from Asian seabass (*Lates calcarifer*) at 1 h and 24 h postmortem were characterized. Significant increases in MFI values and the content of solubilized sarcoplasmic proteins in 24 h PM samples (*p* < 0.05) indicated greater postmortem proteolytic activities as the postmortem duration was extended. On the other hand, myofibrillar and alkaline-soluble proteins within the muscles remained intact. Moreover, the effects of 24 h PM contributed to the reduced expressible water and increased meat tenderization without negative impacts on the springiness and cohesiveness of the cooked Asian seabass meat. Free amino acids related to umami and bitterness were increased in the cooked 24 h PM samples. Overall, the current findings suggested that with proper postmortem storage conditions, an extension of postmortem duration from 1 h to 24 h could contribute to superior technological properties relating to the higher content of umami-tasting amino acids in the epaxial muscle of Asian seabass.

## Figures and Tables

**Table 1:** Technological properties of epaxial muscle of Asian seabass collected at different postmortem timepoints ^1^.

Parameter	1 h PM	24 h PM	*p*-Value
Raw sample			
pH	6.51 ± 0.05 ^b^	6.63 ± 0.16 ^a^	0.011
Surface color			
L*-value	39.31 ± 2.44 ^b^	43.82 ± 4.06 ^a^	<0.001
a*-value	1.57 ± 0.85	2.00 ± 1.19	0.088
b*-value	−3.38 ± 1.15 ^b^	−2.01 ± 1.33 ^a^	0.001
Thawing loss (%)	1.16 ± 0.21	1.62 ± 0.77	0.238
Cooked sample			
pH	6.81 ± 0.05	6.77 ± 0.11	0.242
Surface color			
L*-value	75.55 ± 2.42	74.24 ± 2.47	0.061
a*-value	−0.40 ± 1.21 ^b^	0.56 ± 0.86 ^a^	0.047
b*-value	7.62 ± 1.36	7.09 ± 1.21	0.153
Cooking loss (%)	15.88 ± 5.48	10.56 ± 4.67	0.149
Expressible water (%)	13.83 ± 2.12 ^a^	9.93 ± 2.65 ^b^	0.004
Texture			
Hardness (g)	132.60 ± 30.90 ^a^	104.70 ± 35.56 ^b^	0.047
Springiness	0.52 ± 0.05	0.51 ± 0.12	0.526
Cohesiveness	0.30 ± 0.03	0.27 ± 0.08	0.145
Chewiness (g)	20.85 ± 5.77	15.74 ± 9.30	0.065

^1^ Data are presented in mean ± standard deviation. ^a,b^ Different letters indicate significant difference (*p* < 0.05) calculated using Student’s *t*-test.

**Table 2:** Free amino acids (mg/100 g sample) in cooked epaxial muscle of Asian seabass collected at different postmortem timepoints ^1^.

Amino Acid		MW ^2^	Taste Threshold ^3^	1 h PM	24 h PM	*p*-Value
		(g/mol)	(mg/100 g)			
Sweet-tasting amino acids						
L-alanine	Ala	89.00	106.80	15.92 ± 0.38 ^b^	16.86 ± 0.29 ^a^	0.026
L-glycine	Gly	75.07	187.68	65.55 ± 2.34 ^b^	73.69 ± 0.41 ^a^	0.004
L-proline	Pro	115.13	172.70	40.44 ± 1.48 ^a^	15.82 ± 0.27 ^b^	<0.0001
L-serine	Ser	105.09	262.73	12.73 ± 0.78 ^a^	8.81 ± 0.04 ^b^	0.001
L-threonine	Thr	119.12	416.92	7.56 ± 0.47	7.84 ± 0.09	0.369
Umami-tasting amino acids						
L-aspartic acid	Asp	133.10	53.24	1.42 ± 0.06 ^b^	3.28 ± 0.05 ^a^	<0.0001
L-glutamic acid	Glu	147.13	16.18	19.37 ± 1.00 ^b^	23.40 ± 0.33 ^a^	0.003
Bitter-tasting amino acids						
L-leucine	Leu	131.17	144.29	3.96 ± 0.14 ^b^	8.89 ± 0.10 ^a^	<0.0001
L-isoleucine	Ile	131.17	131.17	3.38 ± 0.05 ^b^	5.71 ± 0.14 ^a^	<0.0001
L-arginine	Arg	174.20	1306.50	17.91 ± 1.25 ^b^	23.98 ± 1.36 ^a^	0.005
L-histidine	His	155.15	698.18	11.96 ± 0.86 ^a^	7.04 ± 0.30 ^b^	0.001
L-valine	Val	117.15	351.45	2.97 ± 0.12 ^b^	5.63 ± 0.05 ^a^	<0.0001
L-phenylalanine	Phe	165.19	743.36	1.74 ± 0.07 ^b^	3.97 ± 0.06 ^a^	<0.0001
L-lysine	Lys	146.19	1169.52	3.79 ± 0.23 ^b^	13.28 ± 0.04 ^a^	<0.0001
L-tyrosine	Tyr	181.19	72.48	1.05 ± 0.09 ^b^	2.95 ± 0.08 ^a^	<0.0001

^1^ Data are presented in mean ± standard deviation. ^2^ MW = molecular weight of amino acid. ^3^ Taste threshold concentrations of amino acids was cited from Rotzoll et al. [31]. ^a,b^ Different letters indicate significant difference (*p* < 0.05) calculated using Student’s *t*-test.

## Data Availability

The original contributions presented in this study are included in the article; further inquiries can be directed to the corresponding authors.
